# Supplementary material for: A Plasmodium knowlesi A1-H.1 transcriptome time course focusing on the late asexual blood stages
Source: PLoS Pathog. 2026 Nov 3;22(11):e1014727. doi: 10.1371/journal.ppat.1014727 (PMC13754235; doi:10.1371/journal.ppat.1014727)
Supplement: Supplementary file 1 [file ppat.1014727.s007.pdf]

| | RBCs counted | iRBCs counted | Parasitaemia | Life stage composition | | | |
|---|---|---|---|---|---|---|---|
| | | | | Ring | Trophozoite | Schizont | Dead schizont |
| **5 hpi** | 6200 | 202 | 3.3% | 98.0% | 0.0% | 0.0% | 2.0% |
| **14 hpi** | 6312 | 225 | 3.6% | 3.6% | 96.4% | 0.0% | 0.0% |
| **20 hpi** | 588 | 564 | 95.9% | 0.2% | 93.1% | 5.5% | 1.2% |
| **24 hpi** | 695 | 642 | 92.3% | 0.3% | 16.0% | 82.6% | 1.1% |
| **27 hpi** | 579 | 512 | 88.4% | 0.8% | 1.0% | 97.7% | 0.0% |
