# Supplementary material for: A Plasmodium knowlesi A1-H.1 transcriptome time course focusing on the late asexual blood stages
Source: PLoS Pathog. 2026 Nov 3;22(11):e1014727. doi: 10.1371/journal.ppat.1014727 (PMC13754235; doi:10.1371/journal.ppat.1014727)
Supplement: Supplementary file 2 [file ppat.1014727.s009.pdf]

		microarray Pk1(A+), rhesus blood			scRNAseq A1-H.1, human blood	
		Lapp et al. 2015			Howick et al. 2019	Sauve et al. 2025
		in vitro I	*in vitro* II	*ex vivo*	*in vitro*	*in vitro*
SICAvar						
Expression >3rd quartile	PKNH_0620500			x	x	x
	PKNH_1021300		NA		x	
Expression >median	PKNH_0514100					
	PKNH_0408100					
	PKNH_0206000	x	x		x	x
	PKNH_0942700				x	x
	PKNH_0408200		NA		x	
	PKNH_0613200					
	PKNH_1443500	x				x
kir						
Expression >3rd quartile	PKNH_1149300			x	x	x
	PKNH_1000600				x	x
	PKNH_0200700					x
Expression >median	PKNH_0806700		NA			x
	PKNH_1454600				x	
