# Supplementary material for: A Plasmodium knowlesi A1-H.1 transcriptome time course focusing on the late asexual blood stages
Source: PLoS Pathog. 2026 Nov 3;22(11):e1014727. doi: 10.1371/journal.ppat.1014727 (PMC13754235; doi:10.1371/journal.ppat.1014727)
Supplement: Supplementary file 3 [file ppat.1014727.s010.pdf]

**Primer**	**Sequence 5'→3'**	**Target**
ap2g3 - F	AGTCAATACACATACATGGGGAGG	3' region *ap2-g*
ap2g3- R	ATACACATTTGGCTACTCGGC	3' region *ap2-g*
ap2g5– F	GTCGCTTATCCAGGAATAGGGAG	5' region *ap2-g*
ap2g5- R	CGTAAAATTCTGTCGGTTCCTATTGG	5' region *ap2-g*
