# Supplementary material for: A Plasmodium knowlesi A1-H.1 transcriptome time course focusing on the late asexual blood stages
Source: PLoS Pathog. 2026 Nov 3;22(11):e1014727. doi: 10.1371/journal.ppat.1014727 (PMC13754235; doi:10.1371/journal.ppat.1014727)
Supplement: Supplementary file 4 [file ppat.1014727.s011.pdf]

| | **Mean Ct 5' *ap2-g* ± SD** | **Mean Ct 3' *ap2-g* ± SD** | **ΔCt ± SD** | **Relative expression 5' vs 3' *ap2-g* (error range)** |
|---|---|---|---|---|
| **20 hpi** | 28.5 ± 0.2 | 21.5 ± 0.1 | 7.0 ± 0.2 | 0.76% (0.65%-0.88%) |
| **24 hpi** | 27.5 ± 0.5 | 21.0 ± 0.2 | 6.5 ± 0.6 | 1.08% (0.71%-1.63%) |
