# Supplementary material for: A Plasmodium knowlesi A1-H.1 transcriptome time course focusing on the late asexual blood stages
Source: PLoS Pathog. 2026 Nov 3;22(11):e1014727. doi: 10.1371/journal.ppat.1014727 (PMC13754235; doi:10.1371/journal.ppat.1014727)
Supplement: Supplementary file 5 [file ppat.1014727.s017.docx]

## We would like to thank the reviewers for their thorough review of our manuscript and for their insightful and constructive comments. We have carefully considered all suggestions and believe that they have substantially improved the manuscript. We have addressed the comments in detail below and have incorporated the suggested analyses and revisions where appropriate. For some major suggestions that would substantially extend the scope of the study, we have not undertaken the requested analyses, in accordance with the revision plan discussed with and agreed upon by the editor. We provide our rationale for these decisions in the responses below in blue. Line numbers refer to the document with track changes.

## Part I – Summary

### Reviewer #1

This manuscript, titled “A Plasmodium knowlesi A1-H.1 transcriptome time course focusing on the late asexual blood stages,” by De Meulenaere and colleagues, presents one of the first bulk RNA-seq time-course analyses of the asexual intraerythrocytic developmental cycle (IDC) of Plasmodium knowlesi A1-H.1 grown in human erythrocytes. The authors profile five IDC stages from tightly synchronized cultures, identify constitutively expressed genes and stage-specific biomarkers, and compare orthologous transcriptional dynamics with P. vivax. As a comparative resource, the study is solid and likely to be useful to the malaria community, especially for researchers using P. knowlesi as a model for P. vivax biology.

That said, the manuscript seems currently stronger as a community resource than as a research article. Much of the work is descriptive and confirmatory. The major conclusions, showing that P. knowlesi A1-H.1 exhibits a temporally regulated “just-in-time” IDC transcriptional program that is conserved across species, are valuable but largely expected. The most original and exciting observation appears in the transcriptional pattern at the gametocyte-specific transcription factor locus, PkAP2-G, where only the 3′ portion of the gene appears to be transcribed, along with detection of an antisense transcript covering the 3′ UTR. Considering that P. knowlesi A1-H.1 is unable to generate gametocytes, this observation could represent an interesting biological feature, but it is currently presented as an intriguing finding rather than a fully validated and developed biological story.

We thank the reviewer for this overall assessment and agree that the manuscript is intended primarily as a community resource. One of our main motivations was to provide an easily accessible transcriptomic resource for the widely used *P. knowlesi* A1-H.1 line. However, we would like to clarify that the principal contribution of this study is not the demonstration of a conserved “just-in-time” transcriptional programme, which we present as a confirmatory analysis consistent with previous work in *Plasmodium* species. Rather, the central focus of the manuscript is the systematic comparison of temporal transcriptional patterns between *P. knowlesi* and *P. vivax*, together with the accompanying web tool. In addition, we identify stage-specific biomarker genes and constitutively expressed genes that we expect will facilitate future experimental studies using the A1-H.1 line.

We agree that the *pkap2-g* expression observations are intriguing. However, fully resolving their biological significance would require substantial additional experimental work beyond the scope of the present study, which aims at transcriptional dynamics during the IDC. We therefore chose to present these observations as an interesting finding arising from the transcriptomic dataset, with the aim of making them available to the community and providing a starting point for future mechanistic investigations. Additional RT-qPCR was now done to further support the *ap2-g* transcriptional results.

### Reviewer #2

This manuscript describes the generation and analysis of a bulk RNAseq dataset for the Plasmodium knowlesi A1-H.1 clone that grows in vitro in human erythrocytes. PkA1-H.1 is increasingly used both as a model for the related and unculturable major human pathogen Plasmodium vivax and as a subject of study in its own right, where high transfection efficiency and the development of effective genetic tools have powered a wave of studies exploring a range of different aspects of malaria parasite biology. A robust transcriptional dataset for this increasingly used model Plasmodium species will therefore be of broad interest and utility to the malaria research community.

Overall the study is very solid – the methods and analysis approaches are clearly described and rational and the manuscript is well written and clear, with extensive and appropriate references to the broader literature. The work is also novel, as while scRNAseq datasets for P. knowlesi A1-H.1 exist, scRNAseq and bulk RNAseq are highly synergistic, and no previous bulk RNAseq data for this human erythrocyte-adapted clone currently exists. The findings are perhaps less novel – several intra-erythrocytic time course transcriptomes from different Plasmodium species have been published over the years using different technologies, and the overall message here is that the P. knowlesi transcriptome is broadly similar to that of other Plasmodium species, with a few interesting differences highlighted particularly in multi-gene families. However, despite the broadly expected nature of the overall themes, the authors have thought carefully about which details are likely to be of most interest to their colleagues, and several of the technical details, such as identification of highly constitutively expressed genes for use in normalisation of qPCR, and identification of stage-specific biomarkers for annotation of scRNAseq datasets, are going to be very useful for many other labs. Overall therefore this is likely to be a significant landmark publication for the field, and the inclusion of a simple to use web tool to access the data only increases its significance. Some further analysis could be considered and technical notes added as outlined below, but these are on the whole minor issues.

We thank the reviewer for this positive assessment.

### Reviewer #3

The authors of this study have generated an interesting time-course dataset of the P. knowlesi A1-H.1 IDC. This is the first such time course for P. knowlesi, using RNA-Seq. They investigate applications of their dataset and provide a tool for accessing it. The strengths of the work lie in its novelty and the in-depth analyses performed. The authors also make a good case that P. knowlesi can be used as a model for P. vivax.

We feel that a deeper comparison between strains of P. knowlesi, the microarray data, and a more comprehensive comparison to 3D7 would make the manuscript more relevant for understanding what is Plasmodium-specific versus P. vivax-specific. The authors claim that this dataset may be transferable to P. vivax experiments, but too different from Pk1(A+) for direct comparison. While they explore compatibility with P. vivax data, they do not do so for Pk1(A+), and we believe the study would be improved by including this comparison. The only think that is missing, would be analysis of ncRNA data.

Overall, the paper is very well written and easy to follow. There are several nice analyses, such as the time-course comparison to P. vivax, accounting for differences in cycle length. All data are publicly available, and it is valuable to be able to query the time-course comparison via a web interface.

We thank the reviewer for this kind assessment of our work. We addressed the comments on the comparison of our dataset to Pk1(A+) vs *P. vivax*, and the analysis of ncRNA, in the section Major Issues.

### Reviewer #4

I enjoyed reading this paper and found the analysis and figures clearly presented. The study represents an important contribution to the malaria field as the first RNAseq time course of P. knowlesi and the authors have performed a thorough analysis of the data. I have a few minor suggestions/questions.

We thank the reviewer for this positive assessment.

## Part II – Major Issues

### Reviewer #1

The main weakness is the limited novelty provided in the manuscript. The manuscript described expected IDC transcriptional waves and broad conservation with P. vivax, which may be useful but not surprising. Many of the conclusions confirm what one would anticipate from previous Plasmodium transcriptomic studies. As a result, the study only provides a useful resource.

We agree that the manuscript is largely descriptive and confirms expected transcriptional patterns. Our intention was to generate a high-quality transcriptomic resource for *P. knowlesi* for the A1-H.1 line and compare it to *P. vivax* transcriptional patterns, as this line is increasingly used as a *P. vivax* model and of broad interest to the malaria research community (see also summary of Reviewer 2). In addition to the dataset itself, we aimed to maximise accessibility by developing an online tool and carefully selecting genes and gene families of broad community interest for visualisation.

The main aim of our dataset as a resource, was stated in following places:
- Abstract: “*This time course dataset provides a reference transcriptome framework for P. knowlesi A1-H.1 and a resource for comparative Plasmodium biology*.”
- Discussion lines 710-713: “*Overall, the P. knowlesi A1-H.1 transcriptome time course presented here provides a resource to advance our understanding of P. knowlesi human infections, and serves as a tool to identify P. vivax orthologous genes with similar expression patterns that can be studied in P. knowlesi line A1-H.1.*”

And we agreed with the editor to further clarify this positioning in the revised manuscript:
- Author summary: we conveyed the message of this dataset as a resource with online tool clearly in the newly added author summary.
- Introduction lines 108-109: “*The resulting dataset provides a reference transcriptome framework for P. knowlesi A1-H.1*.”

A second weakness is the limited temporal resolution. Five time points are useful, but still relatively restricted for a 27-hour IDC, especially when the gene expression profiles are compared to the 48 hours P. vivax profiles. While the authors are cautious about classifications of genes as having similar or dissimilar expression patterns across species, these assignments may still appear somewhat arbitrary. This is important because the authors claim that, of the 3,877 orthologous gene pairs assessed, 75% were classified as having similar temporal expression patterns to P. vivax. This percentage may not be as precise as the authors suggest.

We agree with this point and revised the manuscript to provide a more nuanced interpretation of the proportion of genes classified as having similar or dissimilar expression patterns relative to *P. vivax*:
Line 354-358: “*Since the classification of orthologous gene pairs as similar or dissimilar depends on arbitrarily selected parameters (e.g. temporal shift allowed between orthologous expression patterns) and a limited number of P. knowlesi time points, these similarity percentages should be interpreted as a general indication of similarity between P. vivax and P. knowlesi gene expression rather than as exact values*.”
Lines 587-589 (discussion): “*Approximately three quarters of the P. vivax genes with an orthologue in P. knowlesi ~~(2920/3877)~~ were classified as having expression profiles similar to their P. knowlesi orthologues.*”

In addition, we more clearly highlighted the limited number of time points in the Discussion in lines 578-579 (“*Although this dataset has a relatively limited temporal resolution, comprising five time points with an emphasis on the late asexual stages,…*”) and lines 719-721 (“*Future transcriptome time courses with increased temporal resolution during the ring and trophozoite stages could better capture early asexual-stage transcription*”).

We would also like to clarify that the round figure of 75% was purely coincidental and not a percentage we aimed for beforehand.

A major weakness of this manuscript is that one of the most interesting observation (the unusual PkAP2-G transcription pattern and antisense 3′ UTR transcript), is not followed up and validated experimentally. This is unfortunate as the PkA1-H.1strain seems to be unable to generate sexual parasite forms. The authors rule out some simple explanations, such as gene deletion or promoter mutations. However, without independent validation, this result remains preliminary. If feasible, the authors should validate this observation experimentally. Even modest follow-up would strengthen the manuscript considerably. For example, targeted RT-PCR across the 5′ and 3′ regions, independent confirmation in additional biological replicates and additional strains as well as deeper discussion of possible mechanisms would improve the impact of this manuscript.

We agree that this is a preliminary observation and further emphasised this in the Discussion in lines 664-666 (“*The apparent absence of full-length transcription may result in inefficient production of a complete, functional PkAP2-G protein, and hence hinder sexual conversion, but this hypothesis should be experimentally confirmed in a follow-up study*”) and 721-723 (“*The apparent lack of full-length pkap2-g transcripts should be further validated experimentally to investigate its potential link to the lack of gametocyte production in Pk A1-H.1.*”).

While experimental validation was originally considered beyond the scope of this resource-focused study, we recognise its value. We therefore performed an RT-qPCR on the 20 hpi and 24 hpi samples in which *ap2-g* expression was observed, using one primer set targeting the non-transcribed area of the *ap2-g* CDS (5’ side), and another primer set targeting the transcribed *ap2-g* CDS area (3’ side). Although no reads were detected in the 5’ side by mRNA-seq (vs 248 reads on average in 3’ side), RT-qPCR detected expression in the 5’ side, but at only 0.65%-1.63% of the 3’ side expression. This information was added to the Results in lines 543-546 and Supplementary Tables S4 and S5.

Furthermore, we included an additional biological replicate (A1-H.1, from a different cryopreserved vial than the one used for the time point cultures) in Supplementary Figure S4A in which similar incomplete *pkap2-g* expression can be observed.

### Reviewer #2

1) Comparison with scRNAseq data.

(i) Identification of highly expressed genes at each time point and using them to annotate the previously published scRNAseq data in Figure 4 is of great interest, but the comparison between this new annotation and the original annotation using P. berghei array data, is not taken as far as it could be. Is it possible to quantitate changes in sc stage assignment that would result from using these new biomarkers?

To address this comment, we added 2 new panels to Figure 4 (F, G), showing how cells were differently assigned to hpi time blocks/life stages in our annotation and the *P. berghei*-based annotation, along the pseudotime axis. The most striking difference between our and the *P. berghei*-based annotation is the transition between late schizont and ring stage, which is why we quantitated those changes specifically, and addressed this in lines 230-232: “*Using our biomarker-based annotation, 60% and 21% of cells previously annotated as early ring stages were reassigned to 27 hpi schizonts in the Howick et al., 2019 and Sauve et al., 2025 datasets, respectively*.”

We did not quantify other changes because our selected time points do not correspond directly to the life stages from the *P. berghei*-based annotation (which was based on clustering, not on hpi). For example, it is not clear to which hpi time point the *P. berghei* ‘mid-troph’ annotation would correspond (see Figure 4F). However, we think that the extra panels added to the figure will be helpful for readers to assess the differences between both annotations in a semi-quantitative way.

(ii) Visually the two scRNAseq datasets also look significantly different, with the HIVE data apparently under-represented in very early and very late time point cells – was this highlighted in the original manuscript or is it emphasised more here because of the new biomarkers?

This is a correct observation, which was also described in the original HIVE manuscript (Table S5 from highlights low ring percentages, Figure 4 highlights lower numbers of late schizont stages by showing *DBP* and *NBPX* expression), and matches the observation with our new biomarkers.

(iii) Similarly, does the slightly less smooth biomarker expression curves in the HIVE data (Figure 4E) signal a larger issue with synchrony in this dataset?

HIVE scRNA-seq has limitations compared to 10X, and we do notice that the quality of the HIVE libraries is lower than those of 10X (not in number of transcripts, gene recovery, coverage or mapping; possibly just a larger error on the measured expression levels for HIVE data). As HIVE is a different technology than 10X, certain transcripts might be detected less well. Both aspects can indeed make the biomarker expression curves slightly less smooth (cells are less well ordered along the pseudotime axis). This is mostly visible for the 14 hpi and 20 hpi biomarker curves. Likewise, in their respective UMAP visualisatons, we observe that less cells are highlighted (due to quality or just differences in technology, these sets of biomarkers are less well detected). Thus, the usefulness of the biomarkers depends on the single-cell technology and/or detection limit of these specific genes. We selected them based on their high expression in bulk data, but this doesn’t warrant high expression in single-cell data.

However, we want to stress that HIVE scRNA-seq is highly suitable for sample collections in field studies (as highlighted by Sauve et al. 2025), so both technologies have their own use cases.
We do not believe that there is a large issue with synchrony in the HIVE dataset: as the Reviewer already noticed, the HIVE dataset contains less ring and late schizonts stages, and consequently, the majority of the cells are trophozoites and early-mid schizonts. Since there are so many of these life stages with similar expression patterns, the ordering of these cells on the pseudotime axis by the used algorithms might be noisier. With a more equal amount of all life stages (Sauve et al. applied a conservative filtering that removed some younger cells), the biomarker curves might look better.

(iv) The authors note that several cells that are called as early rings in the original scRNAseq datasets are called as schizonts using the biomarkers – can this be quantitated, how many cells shift assignment?

Addressed in answer to (i).

(v) Overall a more detailed and rigorous comparison of the bulk and scRNAseq data, highlighting any issues with the previously published work, would be helpful – these are widely used as references in the field, so this is a chance to explore limitations and strengths in a way that is broadly useful.

It would be interesting to fully investigate the differences between our bulk RNAseq data, HIVE and 10X sequencing for the *Plasmodium* field, but considering the current goal of giving a use case for the identified biomarkers, this would lead us too far.

However, our biomarkers from bulk RNA-seq reveal differences in previous single-cell datasets (10X, HIVE), as explained in the answer to (iii). To guide researchers towards one of both technologies, and explain better why the HIVE biomarker curves look noisy, we added some additional insights to the Results. Lines 244-2249: “*It can be observed that the biomarkers show less smooth expression along pseudotime for the HIVE dataset (Fig. 4E). This likely reflects a combination of factors, including the lower quality of UMAPs from HIVE scRNA-seq (a platform for use in field studies – (31)), the high proportion of trophozoite and early schizont stages in this dataset, and the lower detection level of some biomarker genes (Fig. 4C).*”

2) Synchrony. Lack of synchrony is a major potential confounder for any blood-stage Plasmodium RNAseq dataset, and the authors have used multiple different technical approaches to ensure that each sample is as tightly synchronised as possible. However in the Discussion they note that there are a small number of dead schizonts present in the 5 hour sample, and advance this as a possible explanation for why some invasion-associated genes are also apparently expressed in early ring stages. They used Guanidine HCL to try to remove these schizonts, but how do they know the parasites are ‘dead’, given PSS was only added to the later time points? Can they calculate the % of these cells present based on counting Giemsa slides? Similarly, do these cells persist at any other time points, and if so, can they be quantitated in the same manner? To be clear, the authors have done everything they can in the system to ensure synchrony and this is not a major concern, but it is important to be transparent about the extent of this as a potential confounder, even if highly limited.

We chose GuHCl over PSS, since PSS only inhibits schizont rupture but does not kill the unruptured schizonts. For the ‘5 hpi flask’, the use of PSS would mean that some schizonts (which often have not burst yet at 5 hpi) would still be alive at the harvest time point.

We extensively tested GuHCl prior to this time course experiment, and could confirm by microscopy that it very effectively kills all schizonts. We also re-checked the 5 hpi smear (made at the time of harvest) and could observe only dead schizonts on the smear.

Since the number of dead schizonts, as well as the number of other life stages than those aimed for, is indeed relevant, we added a new Supplementary Table S1 with an overview of life stage composition per time point.
- 5 hpi smear: we counted 2% of dead schizonts out of all iRBCs (vs 98% of ring stages). This is added to the Results in line 451. No live schizonts were counted on this smear.
- 14 hpi smear: no dead schizonts were observed in 6312 counted RBCs.
- 20 hpi smear: 1.2% dead schizonts out of all iRBCs. This was a Nycodenz-purified smear (unlike 14 hpi or 5 hpi), which allowed to observe more iRBCs, and consequently, increased the chance to observe dead schizonts. Potentially, no dead schizonts were observed in the 14 hpi smear because the parasiteamia was low (3.6%, vs ~90% in the Nycodenz-purified smears).
24 hpi: 1.1% dead schizonts out of all iRBCs
27 hpi: no dead schizonts observed in 579 counted (i)RBCs.

### Reviewer #3

As the data are already generated, we refer here to key analyses rather than new experiments.

As can be seen in Fig. 2A, the RNA-seq library is strand-specific. Further, on the left gene there is clear evidence of ncRNA on the reverse strand of the gene (no splicing on ncRNA, nice splicing on gene). Recently, in P. falciparum biology, the role of ncRNA has gained considerable traction. Would it be possible to detect and analyse ncRNAs in this dataset? Relevant questions could include: are there specific stages with increased antisense transcription? Does this occur primarily in UTRs or across gene bodies?

We thank the reviewer for this suggestion, ncRNA is an important and emerging area, and we made the same observation of antisense transcription in our data. After careful consideration and discussion with the editor regarding the scope of the revision, we decided not to perform this additional analysis because (i) our mRNA-seq library prep protocol made use of poly(A)-based mRNA selection and therefore does not capture all ncRNA types, and (ii) UTR annotations for *P. knowlesi* remain incomplete, limiting robust analysis of antisense transcription. However, we ensured that the availability of the dataset for future studies is clearly stated, and added it as a future study topic in Discussion lines 724-725: “*this dataset could be used to annotate P. knowlesi UTRs, identify poly(A)-containing non-coding RNAs, or …*”.

The authors state that the similarity of P. knowlesi A1-H.1 and Pk1(A+) time courses is “expected to be limited”. In the discussion, they note that a comparison (e.g. of invasion ligand expression) would be “highly informative”. I agree, and suggest that a broader comparison would be even more valuable. Despite the challenges of comparing microarray and RNA-seq data, the authors should investigate and discuss this in more depth to demonstrate that their dataset is indeed substantially better suited for comparison with P. vivax.

As line A1-H.1 has been adapted to grow in human red blood cells (whereas Pk1(A+) is maintained in macaque red blood cells), A1-H.1 is often the preferred model for comparison with *P. vivax* for processes occurring in human erythrocytes. Given the widespread use of A1-H.1 for this purpose, we decided to compare our transcriptome time course to the one of *P. vivax*. This decision was therefore driven by the biological question and the needs of the research community, rather than by the relative suitability of the available transcriptomic datasets.

Although our primary objective was to make a comparison to *P. vivax*, we did consider including a comparison with Pk1(A+) as well, as the comparison to a line growing in rhesus cells could potentially reveal interesting *P. knowlesi*-specific biological mechanisms. However, after careful consideration and consultation with the editor, we decided not to perform this comparison. The currently available Pk1(A+) time course data were generated using microarrays, whereas our dataset was generated by RNA-seq. Direct comparison between these platforms is challenging because of their different read count distributions and dynamic ranges. In addition, the microarray probes were designed using a much older *P. knowlesi* genome annotation, which lacks a substantial number of genes present in the current reference. A comparison to the Pk1(A+) microarray data would represent a substantial extension in both scope and length, and in our view would be more appropriate as a separate study (future study topic added in Discussion lines 724-726: “*this dataset could be used to …, or investigate transcriptional differences between the human-adapted Pk A1-H.1 line and rhesus-blood-adapted clone Pk1(A+)*”).

Deeper analysis:
for some analyses the authors include Pf3D7, while for others they do not. Why not include it more consistently for orthologous comparisons? These data could also be incorporated into the web interface.

*P. falciparum* is not closely related to *P. knowlesi*. ‘Only’ 56% of *P. knowlesi* genes have an orthologue in *P. falciparum* (vs 88% *P. knowlesi* genes have an orthologue in *P. vivax*). This information was added to the Methods in lines 896-897. Due to this limited orthology, we could not update the figures on multigene families or invasion ligands to include *P. falciparum*. Many of the multigene families discussed in our study are absent or poorly represented in *P. falciparum* (*e.g.* no *SICAvar* family, very few *TRAg* genes, and no *kir* genes), and orthologues of invasion ligands are limited. Figure 4 showing ApiAP2 genes (highly conserved within *Plasmodium*) already includes *P. falciparum.*

While the editor agreed with our reasoning regarding the inclusion of Pf3D7 in the manuscript’s analyses, the editor also pointed out that the incorporation of *P. falciparum* in the webtool would be an added value (*e.g.* for resistance markers that are conserved across the 3 species). We agree with this suggestion and thank the reviewer and editor for bringing it to our attention. We have therefore added a new tab to the webtool that allows users to investigate expression patterns of *P. knowlesi-P. falciparum* orthologues: <https://interactive.itg.be/app/pk-orthologue-expression-viewer>.

Additionally, for orthologues that do not share the same temporal expression patterns, are these regulated by the same AP2 factors? This could be an interesting aspect to explore.

ApiAP2 research in *P. knowlesi* is not yet as advanced as in *P. falciparum*, and at this point, *Pk*ApiAP2 motifs are not yet described. Although motifs might be conserved between *P. falciparum* and *P. knowlesi*, this would remain speculation and no conclusive answer could be given.

### Reviewer #4

None.

## Part III – Minor Issues

### Reviewer #1

The manuscript is heavily descriptive across several sections, particularly the multigene-family analyses. The paper would also benefit from a sharper discussion of limitations. These include the limited number of time points, the absence of experimental validation for most interesting results and the fact that comparisons are being made across datasets generated in different contexts and cell cycles.

We addressed the limitations more clearly in the Discussion:
- Limited temporal resolution: lines 578-579: “*Although this dataset has a relatively limited temporal resolution, comprising five time points with an emphasis on the late asexual stages, it allowed us to…*”; lines 719-721: “*Future transcriptome time courses with increased temporal resolution during the ring and trophozoite stages could better capture early asexual-stage transcription.”*
- Limited *ap2-g*experimental validation (additional experimental validation was added based on the reviewer’s suggestion, see Major Issues): lines 664-666: “*The apparent absence of full-length transcription may result in inefficient production of a complete, functional PkAP2-G protein, and hence hinder sexual conversion, but this hypothesis should be experimentally confirmed in a follow-up study*”); lines 721-723 (“*The apparent lack of full-length pkap2-g transcripts should be further validated experimentally to investigate its potential link to the lack of gametocyte production in Pk A1-H.1.*”).
- Comparisons made across datasets generated in different contexts: lines 613-621. This section describes reasons why there might be a shift in timing observed between *P. vivax* and *P. knowlesi* genes: 1) lower synchrony of the *P. vivax* dataset, 2) lab strain vs clinical isolate, 3) IDC lengths. We now conclude the paragraph with this statement: “*Together, these differences in experimental conditions and IDC timing should be considered when interpreting similarities and differences in transcriptional dynamics between P. knowlesi and P. vivax.”*

Furthermore, we revised the Results sections on multigene families, biomarkers and invasion genes to reduce the level of description. As other reviewers asked to add additional information to the sections of biomarkers (+single-cell analysis) and invasion genes, these sections were expanded as well.

### Reviewer #2

1) Figure 1. As noted above, the authors have controlled for synchrony very carefully using Nycodenz tight synchronisation at the outset, Nycodenz purification of late stages at some time points, and Guanidine HCL synchronisation and PSS treatment to prevent re-invasion. Figure 1 therefore rather under-represents the extent to which they have tried to make each sample tightly synchronised, as several of these details (eg Guanidine HCL/PSS treatment) are not annotated in Figuer 1 and are only detailed in the Methods. The overview of the experimental design in Figure 1 could be improved significantly by adding in these details.

We appreciate the reviewer’s attention for our synchronisation efforts, and updated Figure 1 accordingly.

2) Multigene families. The identification of a small number of more highly expressed members of large multi-gene SICAvar and kir gene families is particularly interesting, as it might be presumed that these families, primarily involved in immune evasion, might be largely absent in this in vitro cultured lines. Are the same SICAvar and kir genes identified as the most highly expressed in the previous scRNAseq data? If not, could this be evidence of expression switching during extended in vitro culture?

Generally, the same *SICAvar* and *kir* genes are identified as most highly expressed. To check this, we extracted the 10 most highly expressed *SICAvar* or *kir* genes in the scRNA-seq data from Howick et al. and Sauve et al., by i) annotating cells to a ‘hpi’ time point (see Fig. 4 panels F and G), ii) calculating the median expression value per gene for the cells annotated to every ‘hpi’ time point, and iii) obtain the maximum expression value out of the 5 ‘hpi’ time points for every gene.

Our *SICAvar* gene with the highest expression level (PKNH_0620500) appears in both single-cell datasets as most highly expressed, and our 2 most highly expressed *kir* genes appear in both single-cell datasets as the two most highly expressed *kir* genes. Apart from these top hits, the additional highly expressed *SICAvar* and *kir* genes are not necessarily found back among the 10 top hits in the single-cell datasets (sometimes they are, sometimes not, sometimes only in 1 of both single-cell datasets).

Considering that the top-expressed genes are shared between our dataset and the single-cell datasets, we do not observe direct evidence of expression switching *in vitro*. On the other hand, expression switching is usually not observed under unchanged culture conditions. The single-cell data come from line A1-H.1 as well, and were grown under the same culture conditions (those described by Rob Moon in 2013).

We added this additional analysis to the
- Results (new paragraph, line 309-317)
- New Supplementary Table S3.
- Discussion (lines 695-701): “*Interestingly, the two most highly expressed kir genes (PKNH_1149300, PKNH_1000600) were also the most highly expressed kir genes in the two published Pk A1-H.1 scRNA-seq datasets (30, 31). Likewise, the SICAvar gene with the highest expression level in our dataset (PKNH_0620500) was the most highly expressed SICAvar gene in both scRNA-seq datasets. The consistent predominant expression of these genes across independent A1-H.1 experiments provides no evidence for extensive transcriptional switching of kir and SICAvar genes under A1-H.1 in vitro conditions.*”

Similarly, is it really not possible to compare this data with the previous array-based P. knowlesi H strain data – even to answer the question whether the same SICAvar and kir genes are abundantly expressed in rhesus RBCs?

The main issue with the previously published microarray data (Lapp et al. 2015) is that 36% of the *SICAvar* and 26% of the *kir* genes were not yet present because the probe design was based on an older genome annotation. However, we were lucky that the majority of our highly expressed *SICAvar* and *kir* genes were also detected in the microarray dataset, and we could perform a general comparison. We listed the 10 most highly expressed *SICAvar* and *kir* genes from the microarray datasets (*in vitro* and *ex vivo*), based on the maximal gene expression level out of all sampled time points. The *ex vivo* dataset comes from Pk1(A+) grown in a rhesus macaque (non-splenectomised) followed by short *ex vivo* culturing. The two *in vitro* datasets come from Pk1(A+) adapted to long-term *in vitro* culture in rhesus blood.

This top 10 could be compared to the highly expressed genes in our dataset (>median or >q3 of genome-wide maximal expression levels out of our 5 sampled time points).
- For the *SICAvar* genes, the maximally expressed gene in our dataset also appeared in the top 10 most highly expressed *SICAvar*’s in the Lapp et al. *ex vivo* dataset. Furthermore, two genes that were > genome-wide median (but not >3^rd^ quantile) in our dataset, came back in the top 10 of the Lapp et al. *in vitro* datasets.
- For the *kir* genes, the maximally expressed gene in our dataset also appeared in the top 10 most highly *kir*’s in the Lapp et al. *ex vivo* dataset. Other than that, none of our maximally expressed genes (> genome-wide median) were found in Lapp et al. datasets (*in vitro* or *ex vivo*).

These results are shown in the new Supplementary Table S3, and added to a new Results paragraph (lines 309-317).

### Reviewer #3

The authors correctly state that scRNA-seq has lower sensitivity (on a per-cell basis) compared to bulk RNA-seq, making it “less suitable for generating high-resolution temporal expression profiles”. Whilst there are limitations to scRNA-seq, the temporal resolution it provides at the single-cell level is in fact higher than bulk RNA-seq. I would suggest revisiting and rewording this comparison for accuracy.

We thank the reviewer for pointing out this inaccurate wording. This is now adjusted in lines 98-102: “*While scRNA-seq excels at resolving cellular heterogeneity and capturing dynamic processes, its lower per-cell sequencing depth reduces the sensitivity to detect lowly expressed genes, resulting in sparser gene coverage in temporal expression profiles compared to bulk RNA-seq (32, 33).*”

It would be useful to discuss in more detail the role of P. cynomolgi as a model, including when and why it may be preferable (e.g. liver stages). It may also be worth mentioning that P. knowlesi is comparatively easy to genetically manipulate, and that recent PiggyBac studies on essential genes further support its utility.

We updated the second paragraph of the introduction accordingly (lines 63-73).
We want to clarify that we only mentioned the essential genome study from Oberstaller et al. as it uses line A1-H.1. The gene essentiality study from Elsworth et al., which was published at the same time, used line YH1 which grows in macaque RBCs.

Despite the depletion of human globin mRNA, the authors should discuss the potential impact of residual red blood cell RNA on sequencing depth across samples.

This information was mentioned in the Methods lines 792-794: “*88%-95% of all read pairs per sample mapped to the P. knowlesi genome, with the majority mapping to unique genomic locations (Supplementary Fig. S6). Human contamination was minimal (0.1%-4.2%; Supplementary Fig. S6)*.”.

The % of reads mapping to the human genome is very low, and will therefore not impact the *P. knowlesi* sequencing depth (as can be observed in Figure S6).

The authors should justify their method for selecting biomarker genes for different stages. High expression alone does not necessarily indicate suitability as a biomarker; incorporating a more robust metric may improve the selection.

The reviewer is indeed right that high expression alone would not have been sufficient for biomarker selection. This is why we made use of a combination of fold change and high expression. Firstly, we looked for genes with a fold-change >3 compared to all other life stages. To limit the number of biomarkers, we extracted the top 20 most highly expressed genes from this list (as highly expressed genes have more chance to be detected in scRNA-seq).
In figure 4A, it can be observed that this selection of biomarker genes is robust.

The selection for biomarker genes (in stepwise fashion, for both criteria) was explained in several places:
-the Methods (line 846-849):
“*For each time point of the P. knowlesi time course, 20 biomarker genes were selected. Per time point, genes with a TPM-normalised expression that was >3-fold upregulated compared to every other time point were identified, from which the 20 genes with the highest TPM-normalised expression were selected as biomarkers (Supplementary File S2).*”
-the Results (lines 198-201):
“*Secondly, for each time point, biomarker genes with stage-specific expression were identified based on differential expression (3-fold upregulated compared to every other time point), after which the 20 genes with the highest expression level at that time point were selected (Fig. 4A, Supplementary File S2)*.”
-Figure 4 foot:
“*Per time point, genes with a TPM-normalised expression that was >3-fold upregulated compared to every other time point were identified, from which the 20 genes with the highest TPM-normalised expression at that time point were selected as biomarkers*.”

The authors claim that their biomarker genes can be used to annotate and calibrate future P. knowlesi A1-H.1 and P. vivax single-cell datasets. Whilst the case for P. knowlesi is convincing, no evidence is provided for P. vivax. Given that the manuscript cautions against interspecies comparisons, additional support for this claim would strengthen the argument. It would also be helpful to assess whether these biomarkers fall into the “temporally similar” category defined later in the manuscript.

We agree that it is important to check which *P. knowlesi* biomarkers fall in the ‘temporally similar’ category, and therefore could be used for *P. vivax* (single-cell) data analysis.
Since we selected the biomarkers primarily to be useful for *P. knowlesi* research, 19% of all identified biomarkers do not have an orthologue in *P. vivax*, and for 12% of the biomarkers, the similarity could not be determined because the variability in gene expression over the life cycle was too low for the *P. knowlesi* biomarker or its orthologue(s). 5% of the biomarkers had a *P. vivax* orthologue that was classified as dissimilar. Nonetheless, for every time point, at least 7 biomarkers could be identified with a suitable (‘similar’) *P. vivax* orthologue.
This information is now added to Supplementary File 2, and to the Results in lines 201-203: “*To facilitate their use in P. vivax datasets, orthologues are listed in Supplementary File S2 together with an indication of whether they are suitable to use based on conservation of their temporal expression pattern across the IDC (see later section)*.”

Although the *P. vivax* orthologous biomarkers with a ‘similar’ temporal expression pattern could be applied to *P. vivax* single-cell data, we could not provide evidence for this statement. There is only 1 *P. vivax* single-cell blood stage dataset publicly available (Sà et al., 2020). Here, the asexual life stage annotation was done based on the expression of only 3 genes: MSP3, AMA1 and 60S ribosomal protein L34. This does not allow for a proper comparison of life stage/time point annotation, which is why we changed the concluding sentence of the Results paragraph in lines 251-252 to: “*… calibrate future P. knowlesi A1-H.1 ~~or P. vivax~~ single-cell transcriptome datasets*.”

Additionally, it would be interesting to plot selected marker genes against pseudotime from the scRNA-seq data to assess how well the bulk RNA-seq data are synchronised.

Figure 4 panels D and E show the average expression of the 20 biomarkers per time point. We now added individual biomarker plots supplementary (Supplementary figure S1). In this figure, it can be observed that the majority of the biomarkers selected for each time point, peak at the expected time point in the single-cell data from Howick et al., 2019 (we selected this dataset as it contains a better mixture of all life stages than the Sauve et al. 2025 data, and resulted in higher quality UMAPs).

The relevance of the sicaVAR analysis could be clarified. Whilst high expression is observed (as expected for variant gene families in culture), the absence of immune pressure limits biological interpretation. The authors should discuss how meaningful these observations are under in vitro conditions.
Similarly, whilst the ligand and variable gene expression analyses are interesting, the lack of immune pressure should be acknowledged more explicitly in the discussion, as in vivo RNA-seq would likely provide more biologically relevant insights.

We fully agree with this comment. A concluding text in lines 701-707 was updated to better fit the reviewer’s comment: “*Nevertheless, expression patterns of Pk A1-H.1 multigene families involved in invasion, cytoadherence, immune evasion or other processes subject to host-specific cues, should be interpreted with caution. Their transcription may not fully reflect the regulatory programmes operating during natural infections, making in vivo transcriptomic studies an important complement to the present dataset.*”

In the section “Comparing expression patterns of invasion-associated orthologues between P. knowlesi and P. vivax”, a brief introduction to the temporal expression clusters would improve readability.

We revised this full paragraph (lines 420-461) to improve readability.

The authors classify some genes as having “dissimilar” expression patterns and suggest functional divergence. Evidence supporting that this approach can reliably infer functional differences should be provided.

We agree that differences in temporal expression patterns alone do not demonstrate functional divergence. To our knowledge, little is known about which *P. vivax-P. knowlesi* orthologues are functionally different, since *P. knowlesi* A1-H.1 is a relatively recent model (2013). We have therefore revised the manuscript to clarify that genes classified as having dissimilar expression patterns represent candidates for divergent transcriptional regulation, which may contribute to species-specific biology but requires experimental validation. The main implication of this analysis is not that function can be inferred directly from expression divergence, but rather that orthologues with divergent temporal expression should be interpreted cautiously when using *P. knowlesi* as a model for *P. vivax*.

We already were cautious in statements such as:

- lines 366-368: “*The majority of the orthologous gene pairs show a similar expression pattern in P. vivax and P. knowlesi, suggestive of a similar function and the possibility to extrapolate P. knowlesi experimental findings to P. vivax.”*

- lines 585-587: “*Because expression pattern similarity serves as a proxy for likely functional conservation between both species, this tool enables rapid identification of which P. knowlesi orthologues are suitable to study gene function in P. vivax*.”

And made similar edits in these lines:

- lines 368-370: “*For approximatively 25% of the orthologous gene pairs, P. knowlesi may not be an appropriate P. vivax model because expression patterns, and thus potentially functions, are ~~probably~~ different*.”

- lines 601-605: “*Only PkMSP7A/PvMSP7 (PVP01_1220400), PkTRAg35.4/PvTRAg17 and PkTRAg40.9/PvTRAg10 showed expression patterns classified as ‘dissimilar’, and thus, ~~likely~~ might have a divergent function between P. knowlesi and P. vivax or may reflect uncertainties in orthologue assignment within these multigene families*.”

Furthermore, we noticed that the Results section where we carry out a GO analysis on the dissimilar genes to give an initial clue on differences between *P. vivax* and *P. knowlesi*, can be misleading, and we therefore rephrased lines 370-373: “*Although differences in temporal expression alone do not demonstrate functional divergence, we explored the GO-annotation ~~The function~~ of these ‘dissimilar orthologues’, as it may provide initial insights into ~~could reveal~~ species-specific biological differences*.”

Possibly beyond the main scope, but it would be interesting to briefly explore whether MD, FD, and GD gene orthologues are associated with fragmented AP2-G expression.

We initially explored this, and included a large set of *P. knowlesi* genes orthologous to *P. falciparum* genes associated with gametocytogenesis, male and female development. We compared transcriptional timing and expression level, but could not identify a clear link to the altered *pkap2-g* expression, although a deeper analysis in the future might still reveal novel insights.

Minor spelling and grammar corrections are needed (e.g. “wide-spread” → “widespread”, “parasitaemia's” → “parasitaemias”).

We have corrected the manuscript’s spelling and grammar to the best of our ability.

The authors should comment on why sicaVAR genes, although named “Schizont-Infected Cell Agglutination antigens”, are not predominantly expressed in schizont stages but appear later (e.g. around 27 h) in this dataset.

We generally consider 27 hpi to be part of the schizont stage (although very late schizonts). However, as the heatmap in Figure 5B shows, the majority of the highly expressed *SICAvar* genes peak at 24 hpi, which is the mid-schizont stage in *P. knowlesi*. They often still show expression at 27 hpi late schizonts as well, but this is not where their peak is located.

For two of the highly expressed *SICAvar* genes, we see a peak at 27 hpi – 5 hpi. Ring-stage expression was observed earlier by Lapp et al. (2015), where they found that 38% of the detected *SICAvar* genes were ring-expressed in an *ex vivo* Pk1(A+) culture in rhesus RBCs.

Since our data comes from *in vitro* cultures where cytoadherence has no role, we cannot say whether the non-schizont stage expression we observe for a few *SICAvar* genes has a biological function or not.

The finding of the partially expressed AP2G is intruiging. Did you find any RNA-Seq reads, that had a poly-A sequencing that was not mapped at the end? So, could it be a short transcript with a real end?
We made use of an mRNA-seq library preparation kit relying on oligo-dT magnetic beads to capture mRNA (see Methods). Hence, all reads sequenced will by default have a polyA tail, and thus, the short *ap2-g* transcripts should have a ‘real end’.
Only very few unmapped (soft-clipped) polyA tails can be observed in our mapped read data in this *ap2-g* area, but this is a normal observation also seen for reads mapping to other genes: during library preparation, the captured polyA-RNA is fragmented prior to cDNA conversion. Therefore, only very few reads will still contain the polyA-tail, even though initially all the RNA fragments contained a polyA tail (if not, they were not captured by the beads).

**Methods**
R package versions and any non-default parameters should be fully reported. Some packages (e.g. TopGO, ComplexHeatmap) are currently missing version information.

Versions were added to all tools where this was missing. Non-default parameters are reported.

It should be clarified how multi-mapping reads were handled (ignored, assigned to best hit, or randomly distributed).

Multimapping reads were not ignored, but taken into account in a probabilistic manner: reads mapping to up to 10 loci are all kept in the STAR alignment output (1 randomly is assigned as primary alignment, all others as secondary). If a read maps to >10 loci, it is discarded.

Next, we passed this STAR output to Salmon for TPM normalisation. Salmon estimates the chances of every multimapping read being part of e.g. transcript A or B based on other reads mapping to A and B (no multimappers), and then uses a ‘weighted score’ (based on an iterative EM-algorithm) to split this count over gene A and B (*e.g.* 0.9 to gene A, and 0.1 to gene B). Thus, Salmon ignores how STAR split a multimapping read over primary and secondary alignments, but reanalyses them for a correct ‘distribution’ over the multiple transcripts the read aligns to.

For visualisations in IGV, we used the STAR-mapped files, but removed all multimapping genes (also those assigned as primary alignments), by making use of the STAR mapping quality score.

This was clarified in the Methods lines 790-792: “*STAR was run with default multimapping settings, such that reads mapping to up to 10 genomic loci were retained in the alignment output (one hit is randomly marked as primary, additional hits as secondary)*.”
And lines 802-804: “*Salmon retained multimapping reads and assigned them probabilistically to their compatible transcripts, using an iterative model that estimates transcript abundance.”*
And in lines 796-798 the statement for IGV was updated: *“Coverage tracks displayed in IGV were created with deepTools v3.5.5 (112), and only uniquely mapping primary alignments ~~with a~~ (STAR mapping quality of 255) were included*.”

When using reference genomes, the original publications should be cited. The same applies to all computational tools used.

This was updated now, for reference genomes and tools.

The authors should comment on why differential expression testing was not used for biomarker selection. For example, comparing each time point against all others (as commonly done in scRNA-seq) may provide a more robust framework than a simple 3-fold change threshold.

As described in the Methods, we did use differential expression to select our biomarkers, by comparing 1 time point to all others. We then applied a 3-fold change threshold. A fold-change filter is usually combined with filtering based on p-value, but no statistical testing could be perfomed because our data only consists of a single sample per time point, and replicates are required to estimate biological variance. In scRNA-seq, a ‘1 cluster vs all other clusters’ type of analysis is indeed often done, but 1 cluster still consists of many cells, allowing for statistical testing.

We noticed that we make use of the word ‘upregulated’ which implicitly implies a statistical test. We now rephrased Methods lines 846-849 to: “*Per time point, genes with a TPM-normalised expression that was >3-fold ~~upregulated compared to~~ higher than in every other time point were identified, from which the 20 genes with the highest TPM-normalised expression were selected as biomarkers (Supplementary File S2)*.” (similar changes were entered throughout the Results section on biomarkers and Figure 4 foot).

It is unclear why GO annotations from different PlasmoDB releases were used. This should either be standardised to the latest version or discussed in terms of potential impact on downstream analyses.

They were downloaded at different moment, hence the different PlasmoDB releases. Although we agree with the reviewer that the release version matters, they both are still the same version as the reference genome used (v2; now specified in the Methods), and therefore contain the same genes. Although annotations can change between releases, we never mix up releases within a species (the two different releases are for *P. vivax* and *P. knowlesi*), and more importantly, we never use those two different GO datasets (of different releases) in the same analysis. The *P. knowlesi* GO data (release 67) is used for Figure 2 and Supplementary File S1, the *P. vivax* GO data (release 66) is used for Figure 6. Furthermore, GO enrichment analyses are generally robust, and it is unlikely that changes in GO annotations between releases will strongly alter the outcome.

The authors should explain how the number of tests for Bonferroni correction was determined.

This was now added to the Methods in lines 834-836: “*Bonferroni multiple testing correction was applied to identify significantly enriched GO terms (adjusted p<0.05), with the number of tests corresponding to the number of GO terms tested by topGO after applying the nodeSize = 5 filter*.”

While checking the R code, we noticed that the GO analysis results displayed in figure 6 are accidentally wrong (results from an enrichment analysis using different contrasts were shown in the figure). Figure 6 and its accompanying text (lines 376-377) were therefore updated.

The link to the Sauvé reference (2024) does not appear to work and may need updating (possibly 2025?). Additionally, the phrasing “analysed following the methods of Sauvé et al.” could be simplified. As standard pipelines (e.g. Seurat, Scrublet, SCTransform) were used, referencing these directly may improve clarity.

We thank the reviewer for noticing the inconsistencies with the Sauve reference, which was indeed published 2025 (earlier on bioRxiv). This was updated throughout the manuscript.
We added a better description of how the single-cell data of Sauve et al. were processed in lines 852-856, as this was indeed done using standard pipelines: “*Briefly, reads were mapped with BeeNet v1.1.3 (121) to the P. knowlesi strain H reference genome (PlasmoDB v2 release 60) with systematically extended 3′ UTR annotations. The resulting count matrices were imported into Seurat v5.3 (122), where cells were quality filtered, doublets were identified using Scrublet v0.2.3 (123), and expression data were normalised with SCTransform*.”

The approach to orthologue identification should be clarified. Established methods (e.g. OrthoMCL, OrthoFinder) could have been used. The current approach may appear ad hoc, especially as PlasmoDB definitions are later used for AP2 analyses. A short justification would be helpful.

Initially, we used OrthoMCL, but the main drawback is that OrthoMCL is not suited for multigene families (*e.g.*, almost all *TRAg* genes shown in the manuscript are part of the same OrthoMCL orthology group, while it is possible to find closer 1-to-1 orthologues with high sequence identity). This is why we decided to use BLAST, which is now justified in lines 892-893: “*This BLAST-based approach was particularly useful to identify more 1-to-1 orthologues in multigene families, compared to OrthoMCL*”.

ApiAp2 genes were a specific challenge, where neither BLAST nor OrthoMCL offered a solution, which is why we combined several approaches, as explained in lines 893-896.

Supplementary files should be labelled consistently (e.g. “Supporting Information”), and each table should include a brief legend describing its contents.

Our supplementary data files (excel files) are labelled ‘Supplementary File’ S1 up to S4. Their captions are listed under ‘Supporting information captions’. The supplementary figures and tables are labelled Supplementary Figure/Table S1 etc., and are combined in 1 pdf file called ‘Supporting Information’. Captions of the supplementary figures/tables are included in the pdf file, while under ‘Supporting information captions’, the general description of this Supporting Information pdf can be found (*“Supplementary Figures S1-S6 and Supplementary Tables S1-S4”*).

Possibly, some information got lost or changed during the manuscript submission transfer from bioRxiv to Plos Pathogens, but in the currently submitted revision, this should be solved.

**Figures**

Figure 4: Panels B and C would benefit from labelled colour legends.

A legend was added.

Figure 4: Y-axis labels in panels D and E could be clarified.

New axis labels were added.

Figure 4: Violin plots split by lifecycle stage may improve clarity.

We agree with the reviewer that it was not yet clear how many cells were annotated to which life cycle stage using our biomarkers, or using the previous *P. berghei*-based system. Based on a comment of Reviewer 2, we decided to create panels F and G, using bars coloured by cell-annotation along pseudotime, to visualise this.

It should be stated whether statistical tests were used to compare biomarker expression levels.

No statistical tests were done to compare the biomarker-based life stage annotation to the *P. berghei*-based annotation, because they do not map to each other 1-on-1 (as can be observed in panels F and G). For example, it is not clear to which hpi time point the *P. berghei* ‘mid-troph’ annotation would correspond (see Figure 4F). We selected 5 strict hpi time points, while the *P. berghei* annotation is based on UMAP clustering and not directly connected to time points.

Figure 4: Module scoring could be considered instead of averaged expression for a more robust analysis.

Module scoring would produce a relative enrichment score of the biomarker gene set compared to the expression of matched control genes. This is indeed similar to what we aimed to do, while it also reduces the influence of differences in sequencing depth and overall transcriptional activity of cells. Although this is a good approach, we believe that our simpler approach of averaging the biomarker expression levels is sufficient because we already normalised the data with SCTransform, and the outcome is more directly interpretable compared to module scoring. Although module scoring is the preferred approach for a deeper analysis of biomarker expression levels (*e.g.* to compare average values between time points or datasets), we only aimed to visualise ‘where’ biomarker genes are expressed across the developmental trajectory in a qualitative way.

Figure 7: “Expression” should be specified more clearly (e.g. “scaled normalised expression”).

We updated Figure 7 and all other figures with similar labels.

We noticed that in figure 4A, the data was accidentally not log-transformed, while this is the default practice for heatmap representations. We updated the figure accordingly.

### Reviewer #4

In the orthologs between Pv and Pk, do you see a greater genetic divergence in those genes compared to the similar one eg lower sequences similarity within the coding region or upstream that might explain their different timing?

This is an interesting suggestion that we investigated using the % sequence identity of the protein BLAST results to assess the genetic divergence between Pk-Pv orthologues. % identity was indeed slightly lower for the dissimilar genes. Although this difference is significant (Wilcoxon test p<0.0001), it also is rather small (79.2%+-12.4% vs 81.1%+-12.6%; mean +- stdev), which is why we decided to not report it.

Is the truncated expression of AP2G seen in other Pk expression datasets?

Among publicly available datasets, we have not observed it:
-single-cell data Howick et al. 2019, Sauve et al. 2025: not suitable, as 10x and HIVE scRNA-seq by default only capture reads from the 3′ end of transcripts.
-microarray data Lapp et al. 2015: the used microarray probe targets the 5’ region, where we observed no reads in our dataset. Nevertheless, the microarray data shows *ap2-g* expression under all 3 tested conditions (ex vivo, in vitro I, in vitro II), with expression levels ranging from approximately the first to third quartile of the genome-wide distribution, depending on the dataset. Lapp et al. used line Pk1(A+), a monkey-adapted line which still produces gametocytes.

Additionally, we have other in-house mRNA-seq data of line A1-H.1 (from a different cryopreserved vial than the one used for the time point cultures), which again only shows *ap2-g* expression in its 3’ region. We included the coverage of this additional replicate in Supplementary Figure S4A.

Did you or Novogene perform any additional QC on the RNA samples prior to sequencing, or after sequencing (eg RNA agilent, FastQC, etc)?

Prior to sequencing:
-qubit HS RNA: concentrations are in Table 1
-Agilent Fragment Analyzer RQN value by GenomeScan (the WGS was done by Novogene, the RNA by GenomeScan): was added to Table 1

After sequencing:
-FastQC: no abnormalities observed.

I appreciated how the authors could use their bulk dataset to refine the start point of the IDC in the single cell data. I'm wondering if this resolved start point could help address some of the limitations you raise in the discussion with the bulk data (first paragraph page 19), specifically where you see invasion genes expressed at the early ring time point which could be from dead schizonts. Do you see co-expression of your ring marker genes and invasion genes in the single cell data?

We thank the reviewer for this interesting suggestion. We plotted the expression of invasion ligands with unexpected ring stage expression (Figure 7A clusters 4-6 and *NBPX* genes) over pseudotime for the Howick et al. single-cell data. This is now shown in Supplementary Figure S3. We added this additional analysis to the Results in lines 448-459:

*“Firstly, genes in clusters 5 and 6 and the PkNBPX/PvRBP genes show ring-stage expression in P. knowlesi but not in P. vivax. <…>, visualisation of the expression dynamics of those same genes in P. knowlesi single-cell data shows that they generally do not exhibit ring-stage expression* *(Supplementary Figure S3A-C). Secondly, schizont-stage expression begins slightly later in P. knowlesi for genes in clusters 4 and 5, <…>, and the same can be observed for the PkNBPX/PvRBP family. Single-cell expression dynamics confirm that these P. knowlesi genes are indeed expressed exclusively during the late schizont stage (~27 hpi; Supplementary Figure S3B, D) or during the transition from mid- to late schizonts (24-27 hpi; Supplementary Figure S3A-B).”*

Due to this analysis, we also could remove a speculative part from the Discussion in lines 622-628 (and other part moved to earlier section in Discussion, lines 611-619).
