# Supplementary material for: A Plasmodium knowlesi A1-H.1 transcriptome time course focusing on the late asexual blood stages
Source: PLoS Pathog. 2026 Nov 3;22(11):e1014727. doi: 10.1371/journal.ppat.1014727 (PMC13754235; doi:10.1371/journal.ppat.1014727)
Supplement: Peer review history

# Peer review: Decision Letter 0

**DOI:** 10.1371/journal.ppat.1014727.r001

**Authors:** Margaret Phillips, Elizabeth Winzeler

15 Apr 2026

PPATHOGENS-D-26-00667

A *Plasmodium knowlesi*  A1-H.1 transcriptome time course focusing on the late asexual blood stages

PLOS Pathogens

Dear Dr. De Meulenaere,

Thank you for submitting your manuscript to PLOS Pathogens. After careful consideration, we feel that it has merit but does not fully meet PLOS Pathogens's publication criteria as it currently stands. Therefore, we invite you to submit a revised version of the manuscript that addresses the points raised during the review process.

Please submit your revised manuscript by Jun 14 2026 11:59PM. If you will need more time than this to complete your revisions, please reply to this message or contact the journal office at plospathogens@plos.org. When you're ready to submit your revision, log on to https://www.editorialmanager.com/ppathogens/ and select the 'Submissions Needing Revision' folder to locate your manuscript file.

Please include the following items when submitting your revised manuscript:

* A letter that responds to each point raised by the editor and reviewer(s). You should upload this letter as a separate file labeled 'Response to Reviewers'. This file does not need to include responses to any formatting updates and technical items listed in the 'Journal Requirements' section below.

* A marked-up copy of your manuscript that highlights changes made to the original version. You should upload this as a separate file labeled 'Revised Manuscript with Track Changes'.

* An unmarked version of your revised paper without tracked changes. You should upload this as a separate file labeled 'Manuscript'.

If you would like to make changes to your financial disclosure, competing interests statement, or data availability statement, please make these updates within the submission form at the time of resubmission. Guidelines for resubmitting your figure files are available below the reviewer comments at the end of this letter.

We look forward to receiving your revised manuscript.

Kind regards,

Elizabeth A. Winzeler

Guest Editor

PLOS Pathogens

Margaret Phillips

Section Editor

PLOS Pathogens

Sumita Bhaduri-McIntosh

Editor-in-Chief

PLOS Pathogens

orcid.org/0000-0003-2946-9497

Michael Malim

Editor-in-Chief

PLOS Pathogens

orcid.org/0000-0002-7699-2064

**Journal Requirements:**

If the reviewer comments include a recommendation to cite specific previously published works, please review and evaluate these publications to determine whether they are relevant and should be cited. There is no requirement to cite these works unless the editor has indicated otherwise.

1) We ask that a manuscript source file is provided at Revision. Please upload your manuscript file as a .doc, .docx, .rtf or .tex. If you are providing a .tex file, please upload it under the item type u2018LaTeX Source Fileu2019 and leave your .pdf version as the item type u2018Manuscriptu2019.

2) Please provide an Author Summary. This should appear in your manuscript between the Abstract (if applicable) and the Introduction, and should be 150-200 words long. The aim should be to make your findings accessible to a wide audience that includes both scientists and non-scientists. Sample summaries can be found on our website under Submission Guidelines:

https://journals.plos.org/plospathogens/s/submission-guidelines#loc-parts-of-a-submission

3) Please upload all main figures as separate Figure files in .tif or .eps format. For more information about how to convert and format your figure files please see our guidelines:

https://journals.plos.org/plospathogens/s/figures

4) Some material included in your submission may be copyrighted. According to PLOS's copyright policy, authors who use figures or other material (e.g., graphics, clipart, maps) from another author or copyright holder must demonstrate or obtain permission to publish this material under the Creative Commons Attribution 4.0 International (CC BY 4.0) License used by PLOS journals. Please closely review the details of PLOS's copyright requirements here: PLOS Licenses and Copyright. If you need to request permissions from a copyright holder, you may use the PLOS Content Copyright Permission Form Please respond directly to this email and provide any known details concerning your material's license terms and permissions required for reuse, even if you have not yet obtained copyright permissions or are unsure of your material's copyright compatibility. Once you have responded and addressed all other outstanding technical requirements, you may resubmit your manuscript within Editorial Manager. Potential Copyright Issues:

i) Figure 1. Please confirm whether you drew the images / clip-art within the figure panels by hand. If you did not draw the images, please provide (a) a link to the source of the images or icons and their license / terms of use; or (b) written permission from the copyright holder to publish the images or icons under our CC BY 4.0 license. Alternatively, you may replace the images with open source alternatives. See these open source resources you may use to replace images / clip-art: - https://commons.wikimedia.org - https://openclipart.org/.

**Reviewers' Comments:**

Reviewer's Responses to Questions

**Part I - Summary**

Please use this section to discuss strengths/weaknesses of study, novelty/significance, general execution and scholarship.

Reviewer #1: This manuscript, titled “A Plasmodium knowlesi A1-H.1 transcriptome time course focusing on the late asexual blood stages,” by De Meulenaere and colleagues, presents one of the first bulk RNA-seq time-course analyses of the asexual intraerythrocytic developmental cycle (IDC) of Plasmodium knowlesi A1-H.1 grown in human erythrocytes. The authors profile five IDC stages from tightly synchronized cultures, identify constitutively expressed genes and stage-specific biomarkers, and compare orthologous transcriptional dynamics with P. vivax. As a comparative resource, the study is solid and likely to be useful to the malaria community, especially for researchers using P. knowlesi as a model for P. vivax biology.

That said, the manuscript seems currently stronger as a community resource than as a research article. Much of the work is descriptive and confirmatory. The major conclusions, showing that P. knowlesi A1-H.1 exhibits a temporally regulated “just-in-time” IDC transcriptional program that is conserved across species, are valuable but largely expected. The most original and exciting observation appears in the transcriptional pattern at the gametocyte-specific transcription factor locus, PkAP2-G, where only the 3′ portion of the gene appears to be transcribed, along with detection of an antisense transcript covering the 3′ UTR. Considering that P. knowlesi A1-H.1 is unable to generate gametocytes, this observation could represent an interesting biological feature, but it is currently presented as an intriguing finding rather than a fully validated and developed biological story.

Reviewer #2: This manuscript describes the generation and analysis of a bulk RNAseq dataset for the Plasmodium knowlesi A1-H.1 clone that grows in vitro in human erythrocytes. PkA1-H.1 is increasingly used both as a model for the related and unculturable major human pathogen Plasmodium vivax and as a subject of study in its own right, where high transfection efficiency and the development of effective genetic tools have powered a wave of studies exploring a range of different aspects of malaria parasite biology. A robust transcriptional dataset for this increasingly used model Plasmodium species will therefore be of broad interest and utility to the malaria research community.

Overall the study is very solid – the methods and analysis approaches are clearly described and rational and the manuscript is well written and clear, with extensive and appropriate references to the broader literature. The work is also novel, as while scRNAseq datasets for P. knowlesi A1-H.1 exist, scRNAseq and bulk RNAseq are highly synergistic, and no previous bulk RNAseq data for this human erythrocyte-adapted clone currently exists. The findings are perhaps less novel – several intra-erythrocytic time course transcriptomes from different Plasmodium species have been published over the years using different technologies, and the overall message here is that the P. knowlesi transcriptome is broadly similar to that of other Plasmodium species, with a few interesting differences highlighted particularly in multi-gene families. However, despite the broadly expected nature of the overall themes, the authors have thought carefully about which details are likely to be of most interest to their colleagues, and several of the technical details, such as identification of highly constitutively expressed genes for use in normalisation of qPCR, and identification of stage-specific biomarkers for annotation of scRNAseq datasets, are going to be very useful for many other labs. Overall therefore this is likely to be a significant landmark publication for the field, and the inclusion of a simple to use web tool to access the data only increases its significance. Some further analysis could be considered and technical notes added as outlined below, but these are on the whole minor issues.

Reviewer #3: The authors of this study have generated an interesting time-course dataset of the P. knowlesi A1-H.1 IDC. This is the first such time course for P. knowlesi, using RNA-Seq. They investigate applications of their dataset and provide a tool for accessing it. The strengths of the work lie in its novelty and the in-depth analyses performed. The authors also make a good case that P. knowlesi can be used as a model for P. vivax.

We feel that a deeper comparison between strains of P. knowlesi, the microarray data, and a more comprehensive comparison to 3D7 would make the manuscript more relevant for understanding what is Plasmodium-specific versus P. vivax-specific. The authors claim that this dataset may be transferable to P. vivax experiments, but too different from Pk1(A+) for direct comparison. While they explore compatibility with P. vivax data, they do not do so for Pk1(A+), and we believe the study would be improved by including this comparison. The only think that is missing, would be analysis of ncRNA data.

Overall, the paper is very well written and easy to follow. There are several nice analyses, such as the time-course comparison to P. vivax, accounting for differences in cycle length. All data are publicly available, and it is valuable to be able to query the time-course comparison via a web interface.

**********

**Part II – Major Issues: Key Experiments Required for Acceptance**

Please use this section to detail the key new experiments or modifications of existing experiments that should be absolutely required to validate study conclusions.

Generally, there should be no more than 3 such required experiments or major modifications for a "Major Revision" recommendation. If more than 3 experiments are necessary to validate the study conclusions, then you are encouraged to recommend "Reject".

Reviewer #1: The main weakness is the limited novelty provided in the manuscript. The manuscript described expected IDC transcriptional waves and broad conservation with P. vivax, which may be useful but not surprising. Many of the conclusions confirm what one would anticipate from previous Plasmodium transcriptomic studies. As a result, the study only provides a useful resource.

A second weakness is the limited temporal resolution. Five time points are useful, but still relatively restricted for a 27-hour IDC, especially when the gene expression profiles are compared to the 48 hours P. vivax profiles. While the authors are cautious about classifications of genes as having similar or dissimilar expression patterns across species, these assignments may still appear somewhat arbitrary. This is important because the authors claim that, of the 3,877 orthologous gene pairs assessed, 75% were classified as having similar temporal expression patterns to P. vivax. This percentage may not be as precise as the authors suggest.

A major weakness of this manuscript is that one of the most interesting observation (the unusual PkAP2-G transcription pattern and antisense 3′ UTR transcript), is not followed up and validated experimentally. This is unfortunate as the PkA1-H.1strain seems to be unable to generate sexual parasite forms. The authors rule out some simple explanations, such as gene deletion or promoter mutations. However, without independent validation, this result remains preliminary. If feasible, the authors should validate this observation experimentally. Even modest follow-up would strengthen the manuscript considerably. For example, targeted RT-PCR across the 5′ and 3′ regions, independent confirmation in additional biological replicates and additional strains as well as deeper discussion of possible mechanisms would improve the impact of this manuscript.

Reviewer #2: 1) Comparison with scRNAseq data. Identification of highly expressed genes at each time point and using them to annotate the previously published scRNAseq data in Figure 4 is of great interest, but the comparison between this new annotation and the original annotation using P. berghei array data, is not taken as far as it could be. Is it possible to quantitate changes in sc stage assignment that would result from using these new biomarkers? Visually the two scRNAseq datasets also look significantly different, with the HIVE data apparently under-represented in very early and very late time point cells – was this highlighted in the original manuscript or is it emphasised more here because of the new biomarkers? Similarly, does the slightly less smooth biomarker expression curves in the HIVE data (Figure 4E) signal a larger issue with synchrony in this dataset? The authors note that several cells that are called as early rings in the original scRNAseq datasets are called as schizonts using the biomarkers – can this be quantitated, how many cells shift assignment? Overall a more detailed and rigorous comparison of the bulk and scRNAseq data, highlighting any issues with the previously published work, would be helpful – these are widely used as references in the field, so this is a chance to explore limitations and strengths in a way that is broadly useful.

2) Synchrony. Lack of synchrony is a major potential confounder for any blood-stage Plasmodium RNAseq dataset, and the authors have used multiple different technical approaches to ensure that each sample is as tightly synchronised as possible. However in the Discussion they note that there are a small number of dead schizonts present in the 5 hour sample, and advance this as a possible explanation for why some invasion-associated genes are also apparently expressed in early ring stages. They used Guanidine HCL to try to remove these schizonts, but how do they know the parasites are ‘dead’, given PSS was only added to the later time points? Can they calculate the % of these cells present based on counting Giemsa slides? Similarly, do these cells persist at any other time points, and if so, can they be quantitated in the same manner? To be clear, the authors have done everything they can in the system to ensure synchrony and this is not a major concern, but it is important to be transparent about the extent of this as a potential confounder, even if highly limited.

Reviewer #3: As the data are already generated, we refer here to key analyses rather than new experiments.

As can be seen in Fig. 2A, the RNA-seq library is strand-specific. Further, on the left gene there is clear evidence of ncRNA on the reverse strand of the gene (no splicing on ncRNA, nice splicing on gene). Recently, in P. falciparum biology, the role of ncRNA has gained considerable traction. Would it be possible to detect and analyse ncRNAs in this dataset? Relevant questions could include: are there specific stages with increased antisense transcription? Does this occur primarily in UTRs or across gene bodies?

The authors state that the similarity of P. knowlesi A1-H.1 and Pk1(A+) time courses is “expected to be limited”. In the discussion, they note that a comparison (e.g. of invasion ligand expression) would be “highly informative”. I agree, and suggest that a broader comparison would be even more valuable. Despite the challenges of comparing microarray and RNA-seq data, the authors should investigate and discuss this in more depth to demonstrate that their dataset is indeed substantially better suited for comparison with P. vivax.

Deeper analysis: for some analyses the authors include Pf3D7, while for others they do not. Why not include it more consistently for orthologous comparisons? These data could also be incorporated into the web interface. Additionally, for orthologues that do not share the same temporal expression patterns, are these regulated by the same AP2 factors? This could be an interesting aspect to explore.

**********

**Part III – Minor Issues: Editorial and Data Presentation Modifications**

Please use this section for editorial suggestions as well as relatively minor modifications of existing data that would enhance clarity.

Reviewer #1: The manuscript is heavily descriptive across several sections, particularly the multigene-family analyses. The paper would also benefit from a sharper discussion of limitations. These include the limited number of time points, the absence of experimental validation for most interesting results and the fact that comparisons are being made across datasets generated in different contexts and cell cycles.

Reviewer #2: 1) Figure 1. As noted above, the authors have controlled for synchrony very carefully using Nycodenz tight synchronisation at the outset, Nycodenz purification of late stages at some time points, and Guanidine HCL synchronisation and PSS treatment to prevent re-invasion. Figure 1 therefore rather under-represents the extent to which they have tried to make each sample tightly synchronised, as several of these details (eg Guanidine HCL/PSS treatment) are not annotated in Figuer 1 and are only detailed in the Methods. The overview of the experimental design in Figure 1 could be improved significantly by adding in these details.

2) Multigene families. The identification of a small number of more highly expressed members of large multi-gene SICAvar and kir gene families is particularly interesting, as it might be presumed that these families, primarily involved in immune evasion, might be largely absent in this in vitro cultured lines. Are the same SICAvar and kir genes identified as the most highly expressed in the previous scRNAseq data? If not, could this be evidence of expression switching during extended in vitro culture? Similarly, is it really not possible to compare this data with the previous array-based P. knowlesi H strain data – even to answer the question whether the same SICAvar and kir genes are abundantly expressed in rhesus RBCs?

Reviewer #3: The authors correctly state that scRNA-seq has lower sensitivity (on a per-cell basis) compared to bulk RNA-seq, making it “less suitable for generating high-resolution temporal expression profiles”. Whilst there are limitations to scRNA-seq, the temporal resolution it provides at the single-cell level is in fact higher than bulk RNA-seq. I would suggest revisiting and rewording this comparison for accuracy.

It would be useful to discuss in more detail the role of P. cynomolgi as a model, including when and why it may be preferable (e.g. liver stages). It may also be worth mentioning that P. knowlesi is comparatively easy to genetically manipulate, and that recent PiggyBac studies on essential genes further support its utility.

Despite the depletion of human globin mRNA, the authors should discuss the potential impact of residual red blood cell RNA on sequencing depth across samples.

The authors should justify their method for selecting biomarker genes for different stages. High expression alone does not necessarily indicate suitability as a biomarker; incorporating a more robust metric may improve the selection.

The authors claim that their biomarker genes can be used to annotate and calibrate future P. knowlesi A1-H.1 and P. vivax single-cell datasets. Whilst the case for P. knowlesi is convincing, no evidence is provided for P. vivax. Given that the manuscript cautions against interspecies comparisons, additional support for this claim would strengthen the argument. It would also be helpful to assess whether these biomarkers fall into the “temporally similar” category defined later in the manuscript.

Additionally, it would be interesting to plot selected marker genes against pseudotime from the scRNA-seq data to assess how well the bulk RNA-seq data are synchronised.

The relevance of the sicaVAR analysis could be clarified. Whilst high expression is observed (as expected for variant gene families in culture), the absence of immune pressure limits biological interpretation. The authors should discuss how meaningful these observations are under in vitro conditions.

Similarly, whilst the ligand and variable gene expression analyses are interesting, the lack of immune pressure should be acknowledged more explicitly in the discussion, as in vivo RNA-seq would likely provide more biologically relevant insights.

In the section “Comparing expression patterns of invasion-associated orthologues between P. knowlesi and P. vivax”, a brief introduction to the temporal expression clusters would improve readability.

The authors classify some genes as having “dissimilar” expression patterns and suggest functional divergence. Evidence supporting that this approach can reliably infer functional differences should be provided.

Possibly beyond the main scope, but it would be interesting to briefly explore whether MD, FD, and GD gene orthologues are associated with fragmented AP2-G expression.

Minor spelling and grammar corrections are needed (e.g. “wide-spread” → “widespread”, “parasitaemia's” → “parasitaemias”).

The authors should comment on why sicaVAR genes, although named “Schizont-Infected Cell Agglutination antigens”, are not predominantly expressed in schizont stages but appear later (e.g. around 27 h) in this dataset.

The finding of the partially expressed AP2G is intruiging. Did you find any RNA-Seq reads, that had a poly-A sequencing that was not mapped at the end? So, could it be a short transcript with a real end?

Methods

R package versions and any non-default parameters should be fully reported. Some packages (e.g. TopGO, ComplexHeatmap) are currently missing version information.

It should be clarified how multi-mapping reads were handled (ignored, assigned to best hit, or randomly distributed).

When using reference genomes, the original publications should be cited. The same applies to all computational tools used.

The authors should comment on why differential expression testing was not used for biomarker selection. For example, comparing each time point against all others (as commonly done in scRNA-seq) may provide a more robust framework than a simple 3-fold change threshold.

It is unclear why GO annotations from different PlasmoDB releases were used. This should either be standardised to the latest version or discussed in terms of potential impact on downstream analyses.

The authors should explain how the number of tests for Bonferroni correction was determined.

The link to the Sauvé reference (2024) does not appear to work and may need updating (possibly 2025?). Additionally, the phrasing “analysed following the methods of Sauvé et al.” could be simplified. As standard pipelines (e.g. Seurat, Scrublet, SCTransform) were used, referencing these directly may improve clarity.

The approach to orthologue identification should be clarified. Established methods (e.g. OrthoMCL, OrthoFinder) could have been used. The current approach may appear ad hoc, especially as PlasmoDB definitions are later used for AP2 analyses. A short justification would be helpful.

Supplementary files should be labelled consistently (e.g. “Supporting Information”), and each table should include a brief legend describing its contents.

Figures

Figure 4: Panels B and C would benefit from labelled colour legends.

Figure 4: Y-axis labels in panels D and E could be clarified.

Figure 4: Violin plots split by lifecycle stage may improve clarity.

It should be stated whether statistical tests were used to compare biomarker expression levels.

Figure 4: Module scoring could be considered instead of averaged expression for a more robust analysis.

Figure 7: “Expression” should be specified more clearly (e.g. “scaled normalised expression”).

**********

PLOS authors have the option to publish the peer review history of their article (what does this mean?). If published, this will include your full peer review and any attached files.

If you choose “no”, your identity will remain anonymous but your review may still be made public.

**Do you want your identity to be public for this peer review?**  For information about this choice, including consent withdrawal, please see our Privacy Policy.

Reviewer #1: No

Reviewer #2: **Yes:**  Julian Rayner

Reviewer #3: No

**Figure resubmission:**

While revising your submission, we strongly recommend that you use PLOS’s NAAS tool (https://ngplosjournals.pagemajik.ai/artanalysis) to test your figure files. NAAS can convert your figure files to the TIFF file type and meet basic requirements (such as print size, resolution), or provide you with a report on issues that do not meet our requirements and that NAAS cannot fix.

After uploading your figures to PLOS’s NAAS tool - https://ngplosjournals.pagemajik.ai/artanalysis, NAAS will process the files provided and display the results in the "Uploaded Files" section of the page as the processing is complete. If the uploaded figures meet our requirements (or NAAS is able to fix the files to meet our requirements), the figure will be marked as "fixed" above. If NAAS is unable to fix the files, a red "failed" label will appear above. When NAAS has confirmed that the figure files meet our requirements, please download the file via the download option, and include these NAAS processed figure files when submitting your revised manuscript.

**Reproducibility:**

To enhance the reproducibility of your results, we recommend that authors of applicable studies deposit laboratory protocols in protocols.io, where a protocol can be assigned its own identifier (DOI) such that it can be cited independently in the future. Additionally, PLOS ONE offers an option to publish peer-reviewed clinical study protocols. Read more information on sharing protocols at https://plos.org/protocols?utm_medium=editorial-email&utm_source=authorletters&utm_campaign=protocols

# Author comment: Author response to Decision Letter 1

**DOI:** 10.1371/journal.ppat.1014727.r002

22 Sep 2026

Attachment Submitted filename: Response to Reviewers.docx

# Peer review: Decision Letter 1

**DOI:** 10.1371/journal.ppat.1014727.r003

**Authors:** Margaret Phillips, Elizabeth Winzeler

15 Oct 2026

Dear Dr De Meulenaere,

We are pleased to inform that your manuscript, "A *Plasmodium knowlesi*  A1-H.1 transcriptome time course focusing on the late asexual blood stages", has been editorially accepted for publication at PLOS Pathogens.

Before your manuscript can be formally accepted and sent to production, you will need to complete our formatting changes, which you will receive by email within a week. Please note that your manuscript will not be scheduled for publication until you have made the required changes.

IMPORTANT NOTES

(1) Copyediting and Proofreading: The corresponding author will receive a typeset proof for review, to ensure errors have not been introduced during production. Please review the PDF proof of your manuscript carefully, as this is the last chance to correct any errors. Please note that major changes, or those which affect the scientific understanding of the work, will likely cause delays to the publication date of your manuscript.

(2) Appropriate Figure Files: Please remove all name and figure # text from your figure files. Please also take this time to check that your figures are of high resolution, which will improve the readbility of your figures and help expedite your manuscript's publication. Please note that figures must have been originally created at 300dpi or higher. Do not manually increase the resolution of your files. For instructions on how to properly obtain high quality images, please review our Figure Guidelines, with examples at: http://journals.plos.org/plospathogens/s/figures.

We encourage you to use PACE (http://pace.apexcovantage.com/), a figure-checking tool which helps authors ensure all figures meet PLOS requirements so that figures can publish in the highest quality. PACE is a digital diagnostic and conversion tool for figure files. It will provide information about any failed check(s) and, if able, will automatically convert the figure into an acceptable file that passes quality checks.

To use PACE, you must first register as a user. Then, login and navigate to the UPLOAD tab, where you will find detailed instructions on how to use the tool.

(3) Striking Image: Please upload a striking still image to accompany your article if one is available (you can include a new image or an existing one from within your manuscript). Should your paper be accepted, this image will be considered for our monthly issue image and may also appear on our website to feature your article. Please upload this as a separate file, selecting "striking image" as the file type upon upload. Please also include a separate "Other" file with a caption, including credits and any potential copyright information. Please do not include the caption in the main article file. If your image is from someone other than yourself, please ensure that the artist has read and agreed to the terms and conditions of the Creative Commons Attribution License at http://journals.plos.org/plospathogens/s/content-license. Please note that PLOS cannot publish copyrighted images.

(4) Press Release or Related Media: If you or your institution will be preparing press materials for this manuscript, please inform our press team in advance at plospathogens@plos.org as soon as possible. We ask that you contact us within one week to plan ahead of our fast Production schedule. If you need to know your paper's publication date for related media purposes, you must coordinate with our press team, and your manuscript will remain under a strict press embargo until the publication date and time.

(5) Update your Profile Information: Now that your manuscript has been provisionally accepted, please log into Editorial Manager and update your profile, if needed. Go to http://www.editorialmanager.com/ppathogens, log in, and click on the "Update My Information" link at the top of the page. Please update your user information to ensure an efficient production and billing process.

(6) LaTeX users only: Our staff will ask you to upload a TEX file in addition to the PDF before the paper can be sent to typesetting, so please carefully review our Latex Guidelines http://journals.plos.org/plospathogens/s/latex in the meantime.

Thank you again for supporting Open Access publishing; we are looking forward to publishing your work in PLOS Pathogens.

Best regards,

Elizabeth A. Winzeler

Guest Editor

PLOS Pathogens

Margaret Phillips

Section Editor

PLOS Pathogens

Dominique Soldati-Favre

Editor-in-Chief

PLOS Pathogens

***********************************************************

Reviewer's Responses to Questions

**Part I - Summary**

Please use this section to discuss strengths/weaknesses of study, novelty/significance, general execution and scholarship.<br/><br/>

Reviewer #1: The authors have addressed most of my comments.

Reviewer #2: Nothing further to add - the authors have responded thoughtfully and carefully to all suggestions and added key further details to the manuscript as a result. This will be a highly useful and impactful resource for the field, and the authors should be hugely proud of their efforts.

Reviewer #3: The authors have addressed my comments (also those of the other reviewers in my view). The addition of analyses, like the RT-qPCR for ap2-g, the Pf tab in the web tool and the clarified methods do improve the manuscript. Nothing to add to the previous version of my review. Happy to see this published, just three minor comments.

--------------------

**Part II – Major Issues: Key Experiments Required for Acceptance**

Please use this section to detail the key new experiments or modifications of existing experiments that should be absolutely required to validate study conclusions.<br/><br/>Generally, there should be no more than 3 such required experiments or major modifications for a "Major Revision" recommendation. If more than 3 experiments are necessary to validate the study conclusions, then you are encouraged to recommend "Reject".<br/><br/>

Reviewer #1: (No Response)

Reviewer #2: (No Response)

Reviewer #3: The authors rebuttal two of three points, mostly, after talking to the editor it would be out of focus of the manuscript, which I am happy to accept.

--------------------

**Part III – Minor Issues: Editorial and Data Presentation Modifications**

Please use this section for editorial suggestions as well as relatively minor modifications of existing data that would enhance clarity.<br/><br/>

Reviewer #1: (No Response)

Reviewer #2: (No Response)

Reviewer #3: Just smaller points

- I would add a point that single replicates in time courses is normal in the malaria field.

- Not sure if the "lack of pkap2-g ring-stage expression" (l 607–608) confirms, but rather "is consitent with"... so a bit weaker claim

- Is is really a novel pipeline ? (l. 650)

--------------------

PLOS authors have the option to publish the peer review history of their article (what does this mean?). If published, this will include your full peer review and any attached files.

If you choose “no”, your identity will remain anonymous but your review may still be made public.

**Do you want your identity to be public for this peer review?**  For information about this choice, including consent withdrawal, please see our Privacy Policy.

Reviewer #1: No

Reviewer #2: Yes: Julian Rayner

Reviewer #3: No

# Editor report: Acceptance letter

**DOI:** 10.1371/journal.ppat.1014727.r004

**Authors:** Margaret Phillips, Elizabeth Winzeler

Dear Dr De Meulenaere,

We are delighted to inform you that your manuscript, "A *Plasmodium knowlesi*  A1-H.1 transcriptome time course focusing on the late asexual blood stages," has been formally accepted for publication in PLOS Pathogens.

We have now passed your article onto the PLOS Production Department who will complete the rest of the pre-publication process. All authors will receive a confirmation email upon publication.

Thank you again for supporting open-access publishing; we are looking forward to publishing your work in PLOS Pathogens.

Best regards,

Dominique Soldati-Favre

Editor-in-Chief

PLOS Pathogens
